# College and Hospital Notes

**Published:** 1902-04

**Authors:** 


### COLLEGE AND HOSPITAL NOTES.

THE Brooks Memorial Hospital medical staff has been appointed as follows for the coming year: President, George E. Blackham; vice-president, Nelson G. Richmond; secretary, Walter H. Vosburg.

---

THE State Consumption Hospital at Raybrook is getting under way. Plans for the structure were approved recently by the trustees of the hospital. Ground will be broken about the middle of May and it is expected the hospital will be open for the reception of patients next winter.

---

THE College of Physicians and Surgeons at Baltimore has established post-graduate courses from April 28 to June 9, 1902. The courses are arranged under the following groups: (1) Medicine; (2) surgery; (3) medical and surgical specialties; (4) laboratory courses—(*a*) clinical medicine, (*b*) pathology, (*c*) bacteriology, (*d*) pharmacology.

---

THE Missouri State Medical Association will hold its next annual meeting at St. Joseph, May 20, 21 and 22, 1902, under the presidency of Dr. J. D. Griffith, of Kansas City. A program of interest is in preparation and the meeting promises well for the profession of Missouri.

---

A NEW free dispensary has been opened at the Emergency Hospital for those who are unable to pay for medical treatment.

---

A MARINE hospital for Buffalo is now a certainty. Congress voted an appropriation of $125,000, the President approved the bill at once and gave to Representative Alexander the pen he used in signing. To Mr. Alexander much credit is due for his active interest in pushing this work to a successful conclusion.

---

THE annual banquet of the Alpha Omega Delta fraternity of the Medical College of the University of Buffalo was held March 13, 1902. About 150 were present, including several members of the faculty. Mr. A. W. Hengerer acted as toastmaster. George N. Smith of the senior class spoke on Things in general and the senior class in particular. He dissected that body with a careless hand and laid open the faults of its members. William H. Johnson,' 04, spoke on The fraternity and its relations to the alumni. Dr. Roswell Park made the address of the occasion. His subject was Surgery present, and its relations to the past.

---

THE St. Mary's Infant Asylum and Maternity Hospital, at Buffalo, was made the subject of charges as to its management by Dr. Julius Pohlman at a recent meeting of the Buffalo Academy of Medicine. The Academy authorised the appointment of a committee of investigation, which was afterward named, as follows: Drs. William Warren Potter, Francis E. Fronczak, Thomas F. Dwyer, Henry R. Hopkins and Ernest Wende.

---

THE Syracuse College of Medicine has a four years' course of study. Its facilities for instruction are unusually good. The main building is new, erected in 1896, and was carefully planned and constructed to meet in the most approved manner the requirements of a medical education of the present time. Competent judges pronounce this college unsurpassed in the state.

